# DNA Repair and Cytokines: TGF-β, IL-6, and Thrombopoietin as Different Biomarkers of Radioresistance

**DOI:** 10.3389/fonc.2016.00175

**Published:** 2016-07-22

**Authors:** Lucia Centurione, Francesca B. Aiello

**Affiliations:** ^1^Department of Medicine and Aging Sciences, G. d’Annunzio University of Chieti-Pescara, Chieti, Italy

**Keywords:** radioresistance, cytokines, DNA repair, ATM-dependent DNA-damage response, cancer

## Abstract

Double strand breaks (DSBs) induced by radiotherapy are highly cytotoxic lesions, leading to chromosomal aberrations and cell death. Ataxia-telangiectasia-mutated (ATM)-dependent DNA-damage response, non-homologous end joining, and homologous recombination pathways coordinately contribute to repairing DSBs in higher eukaryotes. It is known that the expression of DSB repair genes is increased in tumors, which is one of the main reasons for radioresistance. The inhibition of DSB repair pathways may be useful to increase tumor cell radiosensitivity and may target stem cell-like cancer cells, known to be the most radioresistant tumor components. Commonly overexpressed in neoplastic cells, cytokines confer radioresistance by promoting proliferation, survival, invasion, and angiogenesis. Unfortunately, tumor irradiation increases the expression of various cytokines displaying these effects, including transforming growth factor-beta and interleukin-6. Recently, the capabilities of these cytokines to support DNA repair pathways and the ATM-dependent DNA response have been demonstrated. Thrombopoietin, essential for megakaryopoiesis and very important for hematopoietic stem cell (HSC) homeostasis, has also been found to promote DNA repair in a highly selective manner. These findings reveal a novel mechanism underlying cytokine-related radioresistance, which may be clinically relevant. Therapies targeting specific cytokines may be used to improve radiosensitivity. Specific inhibitors may be chosen in consideration of different tumor microenvironments. Thrombopoietin may be useful in fending off irradiation-induced loss of HSCs.

## Introduction

Approximately half of cancer patients receive radiation as part of the treatment (1). Radiation sensitivity is influenced by neoplastic cell proliferation or quiescence status, resistance to apoptosis, levels of free-radical scavengers, and the ability to repair highly cytotoxic DNA double strand breaks (DSBs) caused by radiation therapy (RT) (2–4). DSBs are the main contributors to RT-induced cell killing through the formation of chromosomal aberrations that lead to cell death. Improperly repaired DSBs increase genomic instability, chromosomal translocation, and cancer risk (1). Thus, the ability to repair DSBs in cancer cells confers radioresistance, while RT reaching normal cells induces, among other side effects, the development of secondary malignancies. Biological approaches exploiting differences in cellular responses to RT between tumor and normal cells are desirable to specifically radiosensitize tumor cells and protect normal cells.

A role of cytokines in promoting tumorigenesis is recognized as an essential component in all tumors (5). Cytokines promote proliferation, survival, invasion, and angiogenesis, which confer tumor cell radioresistance (6). Although RT induces DNA damage, it also upregulates the expression of various interleukins (IL), including IL-1, IL-6, IL-8, transforming growth factor-beta (TGF-β), and tumor necrosis factor (TNF) (6), which, through the activity of the transcription factor nuclear factor κB (NF- κB), further increases the expression of IL-6 and IL-8 (7). Early studies in mice demonstrated the radioprotective effect of IL-6 (8). High levels of serum IL-6 produced by neoplastic plasma cells and bone marrow stromal cells in multiple myeloma are associated with poor prognosis (9, 10). Lung tumors express IL-6 and the transcription factor Stat3 that mediates IL-6-dependent proliferation and survival effects (11). In these tumors and in squamous esophageal carcinoma, Stat3 overexpression confers radioresistance (11–13). Overexpression of TGF-β is associated with aggressive tumor growth in head and neck cancer and breast and prostate cancer (14, 15). We observed TGF-β expression in all non-small-cell (NSC) lung tumors tested (16). TGF-β inhibitors increase radiosensitivity in breast cancer and glioblastoma in animal experimental models (17, 18). TPO regulates megakaryopoieis and supports hematopoietic stem cell (HSC) quiescence and expansion post-transplantation (19). Importantly, mutations resulting in constitutive activation of the TPO receptor are involved in myeloproliferative neoplasms (19). The effects of TGF-β, IL-6, and TPO on DSB repair (summarized in Table 1) will be the focus of this mini-review.

**Table 1:** **Radioprotective effects induced by TGF-β, IL-6, and TPO**.

	Proliferation	Survival	DSB repair	ATM-DDR	NHEJ	HR
TGF-β^a^	+	+	+	+	+	NA
IL-6^b^	+	+	+	+	NA	NA
TPO^c^	−	−	+	NA	+	−

*^a^TGF-β, radioprotective effects of TGF-β on cancer cells*.

*^b^IL-6, radioprotective effects of IL-6 on cancer cells*.

*^c^TPO, radioprotective effect of TPO on hematopoietic stem cells*.

*ATM-DDR, ATM-dependent DNA-damage response; NHEJ, non-homologous end joining DNA repair; HR, homologous recombination DNA repair; NA, not assessed*.

In mammalian cells, non-homologous end joining (NHEJ) and homologous recombination (HR) pathways repair RT-induced DSBs. They differ in the requirement of a homologous DNA template and in the fidelity of the repair. The HR pathway utilizes DNA sequences of the undamaged chromatid as a template (20, 21). Thus, it is an accurate form of repair that functions in the late S and G2 phase of the cell cycle when an identical sister chromatid is available. The NHEJ pathway promotes direct ligation of the DSBs in all phases of the cell cycle. This is an error-prone mechanism that may result in insertions, deletions, or substitutions at the break sites and translocations, when DSBs from different chromosomes are joined.

Different subtypes of these pathways and the ataxia-telangiectasia-mutated (ATM)-dependent DNA-damage response to DSBs have been thoroughly reviewed elsewhere (20–25). Here, pathways utilized by eukaryotic cells in response to RT will be only briefly summarized.

## NHEJ Pathway

The NHEJ pathway is considered the major repair pathway for DSBs in human cells (26) and is divided into three stages: (1) end detection and tethering, (2) processing, and (3) ligation (22). In the first step, the heterodimeric protein Ku70/Ku80 binds to DSB ends, encircles the DNA duplex, and recruits the DNA protein kinase catalytic subunit (PKcs), a member of the phosphoinositide 3 (PI3)-kinase-like family, which contacts the DNA termini. The binding of PKcs promotes the tethering of the two ends allowing two DNA PKcs molecules to interact across DSBs in a “synaptic complex.” This promotes the phosphorylation in “trans” across the DSB and the autophosphorylation of PKcs (20) which induce conformational changes and improve the access of processing proteins and their functions (22). In the second phase, non-ligatable ends are processed to remove blocking end groups and damaged DNA, or to fill in gaps, thus, this is an error-prone process. Different enzymes are required; the most important is the NHEJ-specific nuclease Artemis, whose activity is regulated by PKcs and ATM kinase-mediated phosphorylation (22, 26). Other enzymes, also involved in base excision and nucleotide excision DNA repair pathways, participate in processing, indicating that cells have evolved to a coordinated cascade of events to be protected by DNA damage. These include the phosphatase polynucleotide kinase and exonucleases Exo1 and WRN (21, 22). WRN is mutated in Werner syndrome, characterized by premature aging and genomic instability (27). The nucleotide gaps are repaired by two members of the X family polymerases, pol λ and pol μ.

Finally, ligation of DNA ends is carried out by ligase IV, bound to the scaffolding X-ray cross-complementing protein (XRCC)4, which stabilizes the ligase and stimulates its activity (20–22).

The XRCC4-like factor (XLF) is required to promote ligation of blunt ends and mismatched non-cohesive ends, and to re-adenylate the ligase, a necessary step for ligation (22). Mutations inactivating XLF or ligase IV are associated with growth retardation, radiation sensitivity, and immunodeficiency (28, 29). Noteworthy, the NHEJ pathway is critical in the repair of physiological DSBs created during immunoglobulin V(D)J recombination and class switch recombination. Thus, patients lacking normal NHEJ are sensitive to radiation and immunodeficiency (30).

## HR Pathways

Homologous recombination pathways require undamaged homologous DNA to repair DSBs. In general, HR can also be divided into three stages: presynapsis, synapsis, and postsynapsis (21). During presynapsis, an extensive and complex 5′ to 3′ resection of broken DNA ends occurs to generate 3′ ended single strand DNA.

The heterotrimeric complex MRN formed by Mre11, NBS 1, and Rad 50 cooperates with the C-terminal binding protein interacting protein (CtIp) to remove about 100 nt (23). MRN is also an initial DSB sensor and unwinds DNA ends (25, 31). Breast cancer-associated protein (BRCA)1 is recruited at this site and regulates this step activating end resection and promoting the activity of CtIp (1).

In the second stage, the heterotrimeric replication protein A (RPA), also involved in the nucleotide excision repair pathway (21), binds to single strand tails preventing internal base pairing (23). Then, RPA is replaced by Rad 51 recombinase in conjunction with BRCA2 and a group of proteins known as Rad 51 paralogs required for RPA replacement (1) (Rad 51B, C, and D, XRCC2, and XRCC3), which yield the Rad51 nucleoprotein filament (1, 21, 23). This filament invades the double strand DNA molecule to search sequence homology and forms a structure termed displacement loop (D-loop). Immunofluorescence staining detects Rad51 nucleoproteins as distinct subnuclear foci (23). Following Rad51 removal from the 3′ end to reveal the 3′-OH group, necessary for priming, DNA synthesis starts elongating the invading strand and forming a cross-shaped structure: the Holliday junction (1, 21, 23). HR repair can proceed differently from this point (1). In the synthesis-dependent strand-annealing (SDSA) repair, after DNA synthesis, performed by polymerase δ, the new DNA strand is displaced and re-ligated with the original DNA, thus, SDSA repair is not associated with crossover. Alternatively, in the double strand break repair (DSBR), two independent strand invasions from both DSB ends are followed by simultaneous DNA synthesis (performed by polymerase η), and generate a double Holliday junction (1, 23). Specific enzymes cleave this junction, and depending on which pair of strands is cut the DSBR pathway can lead to a crossover or non-crossover outcome (1, 21, 23).

## ATM-Dependent DNA-Damage Response

In response to DNA damage, including RT, eukaryotic cells activate cell cycle checkpoints: they arrest the cell cycle allowing DNA repair or triggering apoptosis if repair is impossible (25). Kinase-dependent signaling networks regulate checkpoint activation. In parallel to promoting cell cycle arrest, checkpoint signaling mediates the recruitment of DNA repair pathways (25). ATM kinase, containing a PI3 kinase-like sequence, is encoded by the ATM gene, mutated in patients affected by ataxia-telangiectasia (24, 32), whose cells exhibit decreased survival and increased radiation sensitivity (24). ATM signaling is induced by DSBs and also by chromatin perturbations that do not directly cause DSBs (33). ATM exists as an inactive multimer that dissociates into active monomers upon a conformational change associated with autophosphorylation and acetylation by the acetyl transferase Tip 60, which binds the above-mentioned MRN complex (25). MRN recruits ATM at the DSB sites crucially enhancing its activity (31, 34). The minor histone H2A variant contributes to the suppression of genomic instability preventing the separation of cleaved DNA strands (35). ATM phosphorylates H2AX histones surrounding DSBs and a multitude of substrates, including the MRN complex (25). Substrates of ATM also include the checkpoint kinase (CHK)2, CHK1, and p53 (25, 36). Activation of CHK1 and CHK2 contributes to cell cycle arrest at the G1/S and G2/M phases of the cell cycle (25). CHK2 also promotes p53-dependent and -independent apoptosis pathways (37). Thus, ATM activity regulates cell cycle arrest (CHK1, CHK2, p53), apoptosis (p53 and CHK2), and DNA repair (MRN complex and H2AX). H2AX phosphorylation on serine 139 (γH2AX) can be visualized by immunofluorescence as discrete spots or foci or by Western blot analysis. Detection of γH2AX is widely used as an indicator of the incidence of DSBs (38).

## TGF-β, IL-6, and TPO Differently Influence Radioresistance and DNA Repair

**Figure 1:**
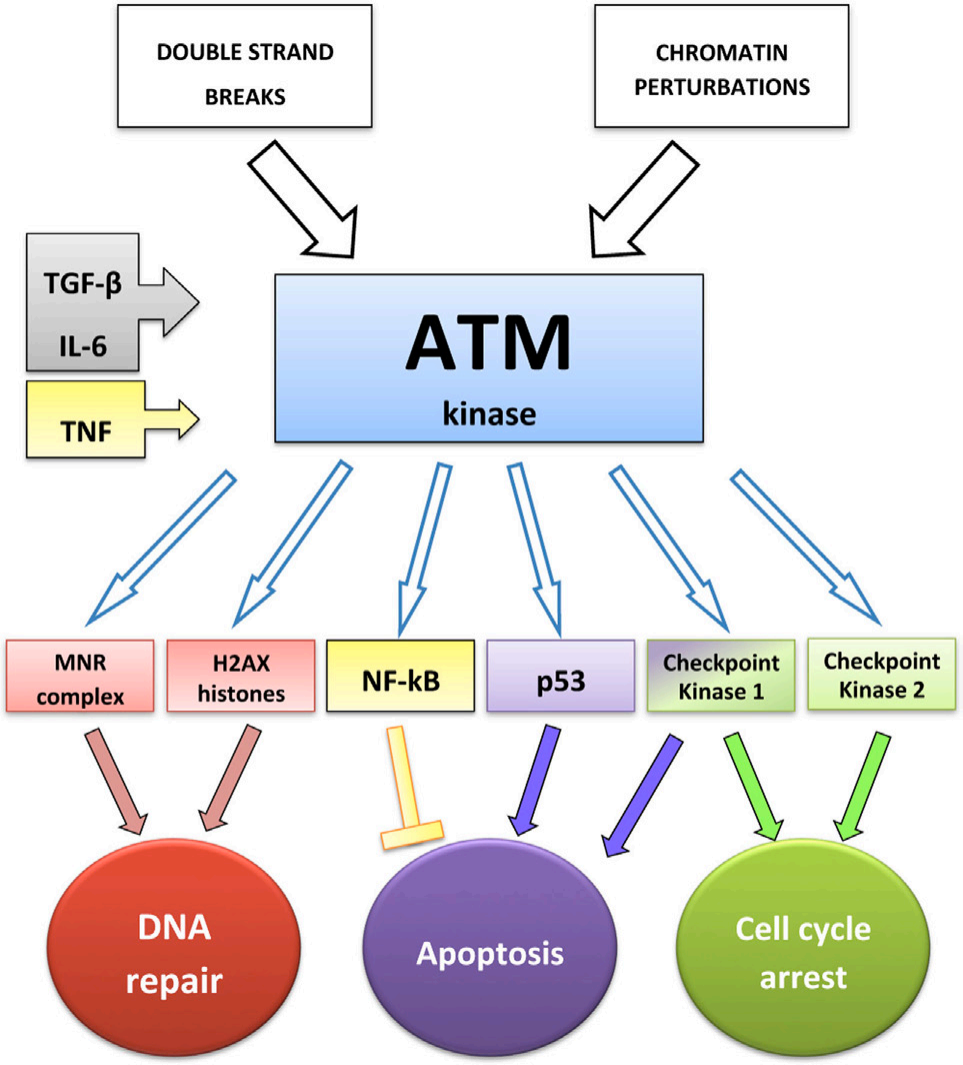
**ATM-dependent DNA-damage response: interactions between ATM and cytokines**. TGF-β increases the level of phospho-ATM (43), IL-6 increases the expression of both ATM and phospho-ATM (45), and TNF induces ATM activation, required to stimulate NF-κB transcriptional activity (46).

Transforming growth factor-beta is a pleiotropic cytokine that regulates proliferation, angiogenesis, and immune responses (39). In normal epithelial cells, it negatively regulates cell cycle progression by activating cyclin-dependent kinase inhibitors, such as p15 and p21 (40). By contrast, it promotes cancer progression and metastasis by a variety of mechanisms, including induction of angiogenesis, cell motility and invasion, and repression of the immune system (39). *In vitro* studies, performed in 2006 showed that the ATM-dependent DNA-damage response to irradiation was impaired in TGFβ-deficient murine mammary cells (41). Subsequently, in human breast cancer cell lines, it was shown that inhibition of TGF-β signaling using the TGF-β type I receptor kinase inhibitor Ly 364947 decreased the clonogenic cell growth prior to irradiation, and blocked irradiation-induced γH2AX foci formation and p53 phosphorylation (18). Moreover, an anti-TGF-β antibody decreased the number of irradiation-induced γH2AX foci and the growth of neoplastic cells injected in immunocompromised mice (18). Similar results were obtained by different laboratories using TGF-β type I receptor kinase inhibitors and glioblastoma murine and human cell lines, and in murine experimental models (17, 42). In addition, an inhibitor was shown to decrease growth and apoptosis of glioblastoma cancer stem cell-like cells (CSCLC), as well as tumor invasion and angiogenesis (42). In murine prostate cancer, it was confirmed that irradiation increased TGF-β expression while its inhibition by a silencing vector increased the level of nuclear phospho-ATM (Figure 1) and the number of nuclear γH2AX foci (43). Recently, in human epidermoid carcinoma cells and in embryonic kidney cells, it was demonstrated that TGF-β pre-treatment not only protected the cell lines from irradiation-induced apoptosis and decreased the amount of nuclear γ-H2AX foci but also increased the expression of ligase IV and promoted the nuclear retention of Ku70/Ku80, ligase IV, and XRCC4 (44). SMAD proteins are intracellular mediators of TGF-β signaling. TGF-β stimulation leads to phosphorylation of SMAD2 and SMAD3, which form complexes with SMAD4 and regulate the transcription of target genes in the nucleus (39). RNA silencing of SMAD2/3 proteins confirmed that ligase IV levels depended on canonical SMAD-dependent signaling. Importantly, ligase IV RNA silencing decreased TGF-β-induced protection against irradiation, underlining the important role of NHEJ repair (44). These data indicate that TGF-β increases radioresistance by multiple mechanisms, including effects on DNA repair, and suggest that specific inhibitors administered before RT might improve radiosensitivity.

Interleukin-6 is a multifunctional cytokine involved in inflammatory processes; it stimulates acute phase protein synthesis, hematopoiesis, survival, and cell growth (12). It can cross the blood–brain barrier and the resulting synthesis of prostaglandin E2 in the hypothalamus changes the body temperature set point (12). Stat3 is the critical regulator of IL-6-dependent cell growth, differentiation, and survival signals. It promotes the transcription of pro-survival regulatory genes: c-myc, B-cell lymphoma (Bcl)-extra large (Bcl-XL), and myeloid cell leukemia 1 (Mcl-1) anti-apoptotic genes, and binds p53 inhibiting its function (12, 13). Transfection of dominant-negative Stat3 abolishes the pro-survival effect of IL-6 (47). IL-6 is involved in proliferation, survival, and differentiation of almost all tumors studied, and is overexpressed in multiple myeloma, oral squamous carcinoma, and in breast, ovarian, prostate, endometrial, colorectal, renal, and lung cancers (12).

Previous studies showed that the administration of anti-IL6 antibody in mice enhanced radiation-induced mortality (8). Other authors confirmed that irradiation enhanced IL-6 expression and showed that the increased growth and angiogenesis of murine hormone resistant versus hormone sensitive prostate cancer cells was attributable to higher IL-6 production (48). It has recently been demonstrated using human NSC lung cancer cell lines that following irradiation CD133^+^ CSCL-like cells proliferated and survived better than CD133^−^ cells. Silencing of IL-6 reduced proliferation and survival in both groups of cells. IL-6 silencing in CD133^+^ cells resulted in a higher number of DSBs compared with CD133^+^ non-silenced cells 3 h after irradiation, indicating a difference in DNA repair. By contrast, no difference was observed between IL-6 silenced and non-silenced CD133^−^ cells. The expression level of ATM, phosphorylated ATM, CHK2, and phosphorylated p53 was lower in IL-6 silenced CD133^+^ cells than in non-silenced CD133^+^ cells, whereas no difference was found between CD133^−^ silenced and non-silenced cells. IL-6 upregulated the transcription of ATM (Figure 1) and, as expected, the expression of anti-apoptotic genes, such as Bcl-2 and Mcl-1 (45). These data indicate that IL-6 specifically affects the DNA-damage response in CSCL cells and suggest the hypothesis of other effects on DNA repair pathways. The evidence that IL-6 induces c-myc (49) lends support to this hypothesis. Radioresistance of nasopharyngeal carcinoma cells is dependent on c-myc-mediated overexpression of CHK1 and CHK2 genes, which display c-myc binding sites on their promoters (48). Moreover, c-myc transcriptional activity promotes NHEJ repair (50, 51), and its silencing in irradiated embryonal rhabdomyosarcoma cells increases the number of γH2AX foci and decreases the expression of DNA PKcs, Ku/70, and RAD51 (51). IL-6-targeted biological therapies are available. The human–mouse chimeric monoclonal antibody CNTO 328 has shown promising results in phase II clinical trials concerning patients with ovarian and renal cancers (12). Patients with multicentric Castleman disease treated with this antibody as a single agent showed high rates of clinical response (52). In addition, clinical trials are ongoing in ovarian cancer patients utilizing the humanized anti-IL-6 receptor monoclonal antibody Tocilizumab in combination with chemotherapy (53).

Myelosuppression and loss of HSCs are important side effects of RT. TPO, essential for megakaryopoiesis, increases proliferation, survival, quiescence, and expansion post-transplantation of HSCs (19). TPO receptor-deficient mice exhibit 10–20% of the normal HSC number. TPO has been shown to exhibit important and selective DNA repair promoting activity (54). HSCs from TPO receptor-deficient mice were more radiosensitive and exhibited decreased DSB repair than cells from wild-type mice. Removal of TPO but not of stem cell factor or Fms-related tyrosine kinase 3 ligand from the medium impaired DSB repair in normal HSC, underlining TPO specificity. Importantly, TPO increased PKcs phosphorylation and promoted NHEJ repair. The number of irradiation-induced Rad51 foci was not modified, suggesting that TPO had no effect on HR repair. HSCs from TPO-receptor-deficient mice showed genomic instability after irradiation, whereas TPO treatment of normal HSC before irradiation protected from genomic instability and improved HSC reconstitution capacity in secondary transplants. Surprisingly, TPO did not modify the cell cycle or survival of irradiated HSCs (54).

Other cytokines support tumor radioresistance, however, whether they promote DNA repair is unknown. These cytokines produced by neoplastic cells and in response to RT include IL-1, IL-8, and TNF (6). The role of IL-1 in enhancing radioresistance was known more than 20 years ago (55). IL-1 activates NF-κB, which mediates the expression of more than 200 genes promoting survival proliferation, invasion, and radioresistance (6). Interestingly, IL-1 may promote DNA repair, since it induces TGF-β expression (56, 57).

Interleukin-8, a pro-inflammatory chemokine enhances proliferation and survival of endothelial cells, leading to neoangiogenesis (58). High levels of IL-8 correlate with a poor prognosis in hepatocarcinoma, colon, and nasopharyngeal cancer (58, 59). In prostate cancer cells and in nasopharyngeal carcinoma, high IL-8 expression confers radioresistance (59, 60), mediated, in nasopharyngeal cancer, by PI3 kinase and Stat3-dependent signaling pathways. Importantly, a Stat3 inhibitor inhibited radiosensitivity (59, 61).

Tumor necrosis factor, a pro-inflammatory cytokine with potent antitumor effects binds to two structurally related but functionally distinct receptors, TNF receptors 1 and 2. Binding to these receptors initiates a complex array of signaling pathways (62, 63), including one that induce activation of NF-κB, leading to cell survival, while the other, through regulation of Fas-associated protein with death domain, can lead to apoptosis (62). Therefore, TNF-α affects radioresistance in a complex manner, cell type dependent, mainly influencing cell survival (62, 63). For instance, in human neuroblastoma cells TNF-α expression induced by RT resulted in sustained NF-κB activation, survival advantage, and radioprotection (64). The ATM kinase activity can promote NF-κB activation following various genotoxic stimuli, including irradiation (46, 65). In the human A549 lung epithelial cell line, TNF induced simultaneously DSBs due to free-radical formation and NF-κB activation (66). Interestingly, NF-κB activation depended on the activation of ATM, which was an unexpected nuclear damage response signal, activated by TNF (46, 65) (Figure 1). NF-κB inhibitors may represent novel radiosensitizing strategies targeting both TNF pro-survival signals and NF-κB-mediated IL-6 and IL-8 production induced by TNF (7, 62).

## Conclusion

The recently demonstrated activity of cytokines on DNA repair pathways may be clinically relevant, suggesting that therapies targeting cytokines could be employed to improve the results of RT in very different contexts. TGF-β and IL-6 confer radioresistance using multiple mechanisms that may be desirable to counteract with specific inhibitors before RT administration. It should be taken into account that TGF-β effects on normal versus neoplastic cells are different, and sometimes opposite, whereas IL-6 has similar effects on normal and neoplastic cells.

Moreover, the different biological behavior of different tumors in terms of different cytokine overexpression should also be considered to obtain specific therapies.

TPO-induced DNA repair activity in irradiated HSCs appears impressively selective, thus, a short treatment to avoid side effects (67) may be useful to protect HSCs before irradiation in solid tumors.

## Author Contributions

All authors listed, have made substantial, direct and intellectual contribution to the work, and approved it for publication.

## Conflict of Interest Statement

The authors declare that the research was conducted in the absence of any commercial or financial relationships that could be construed as a potential conflict of interest.
